# 1-(2,6-Dichloro­benzo­yl)-3-(3,5-dichloro­phen­yl)thio­urea

**DOI:** 10.1107/S1600536808042736

**Published:** 2008-12-20

**Authors:** M. Khawar Rauf, Michael Bolte, Amin Badshah

**Affiliations:** a Department of Chemistry, Quaid-i-Azam University, Islamabad 45320, Pakistan; b Institut für Anorganische Chemie, J. W. Goethe-Universität Frankfurt, Max-von-Laue-Strasse 7, 60438 Frankfurt/Main, Germany

## Abstract

The crystal structure of the title compound, C_14_H_8_Cl_4_N_2_OS, is composed of discrete mol­ecules with bond lengths and angles quite typical for thio­urea compounds of this class. The plane containing the central SONNCC atom set subtends a dihedral angle of 31.47 (3)° with the benzene ring. An intra­molecular N—H⋯O hydrogen bond stabilizes the mol­ecular conformation and the mol­ecules form centrosymmetric dimers *via* inter­molecular N—H⋯S hydrogen bonds.

## Related literature

For general background, see: Upadlgaya & Srivastava (1982 ▶); Wegner *et al.* (1986 ▶); Krishnamurthy *et al.* (1999 ▶). For related structures, see: Khawar Rauf *et al.* (2006*a* ▶, 2007 ▶). For a description of the Cambridge Structural Database, see: Allen (2002 ▶). For bond lengths and angles in *N*,*N′*-disubstituted thio­urea compounds, see: Arslan *et al.* (2004 ▶); Khawar Rauf *et al.* (2006*b* ▶); Yamin & Yusof, (2003 ▶).
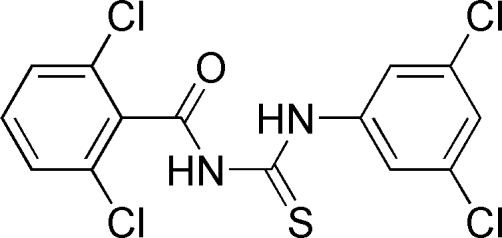


## Experimental

### 

#### Crystal data

C_14_H_8_Cl_4_N_2_OS
*M* *_r_* = 394.08
Monoclinic, 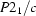

*a* = 14.7737 (13) Å
*b* = 10.3744 (6) Å
*c* = 10.6935 (11) Å
β = 97.250 (7)°
*V* = 1625.9 (2) Å^3^
*Z* = 4
Mo *K*α radiation
μ = 0.86 mm^−1^
*T* = 173 (2) K
0.42 × 0.38 × 0.21 mm

#### Data collection

Stoe IPDSII two-circle diffractometer
Absorption correction: multi-scan (*MULABS*; Spek, 2003 ▶; Blessing, 1995 ▶) *T*_min_ = 0.715, *T*_max_ = 0.841
12823 measured reflections
3724 independent reflections
3306 reflections with *I* > 2σ(*I*)
*R*_int_ = 0.046

#### Refinement

*R*[*F* ^2^ > 2σ(*F* ^2^)] = 0.032
*wR*(*F* ^2^) = 0.087
*S* = 1.05
3724 reflections
208 parameters
H atoms treated by a mixture of independent and constrained refinement
Δρ_max_ = 0.44 e Å^−3^
Δρ_min_ = −0.36 e Å^−3^

### 

Data collection: *X-AREA* (Stoe & Cie, 2001 ▶); cell refinement: *X-AREA*; data reduction: *X-AREA*; program(s) used to solve structure: *SHELXS97* (Sheldrick, 2008 ▶); program(s) used to refine structure: *SHELXL97* (Sheldrick, 2008 ▶); molecular graphics: *XP* in *SHELXTL-Plus* (Sheldrick, 2008 ▶); software used to prepare material for publication: *SHELXL97*.

## Supplementary Material

Crystal structure: contains datablocks I, global. DOI: 10.1107/S1600536808042736/fl2225sup1.cif

Structure factors: contains datablocks I. DOI: 10.1107/S1600536808042736/fl2225Isup2.hkl

Additional supplementary materials:  crystallographic information; 3D view; checkCIF report

## Figures and Tables

**Table 1:** Hydrogen-bond geometry (Å, °)

*D*—H⋯*A*	*D*—H	H⋯*A*	*D*⋯*A*	*D*—H⋯*A*
N2—H2⋯O1	0.91 (2)	1.89 (2)	2.6581 (17)	141 (2)
N1—H1⋯S1^i^	0.82 (2)	2.57 (2)	3.3653 (14)	163 (2)

Symmetry code: (i) 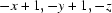.

**Fig. 1.**
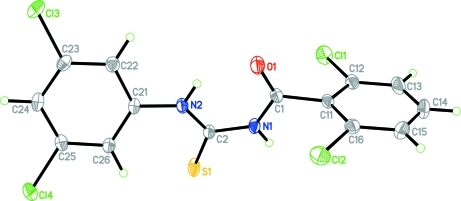
Molecular structure of (I) showing atom numbering scheme. Displacement ellipsoids are drawn at the 50% probability level.

**Fig. 2.**
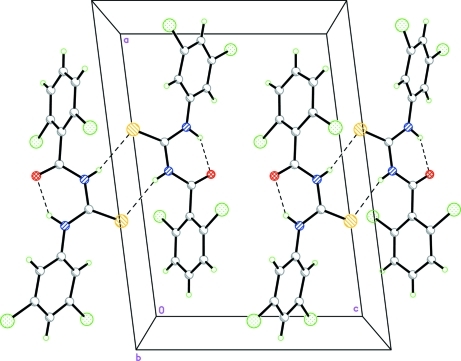
Partial packing diagram of (I) with view onto the ac plane. Hydrogen bonds shown as dashed lines.

## supplementary crystallographic information

### Comment

Aliphatic and acylthioureas are well known for their fungicidal,antiviral, pesticidal and plant-growth regulating activities (Upadlgaya & Srivastava, 1982; Wegner *et al.*, 1986). Symmetrical and unsymmetrical thioureas have shown antifungal activity against the plant pathogens *Pyricularia oryzae* and *Drechslera oryzae* (Krishnamurthy *et al.*, 1999). The background to this study has been set out in our previous work on the structural and biological chemistry of chloro substituted *N*,*N'*-disubstituted thioureas (Khawar Rauf *et al.*, 2006*a*; 2007).The biological studies of these thiourea derivatives are under investigation. Herein, as a continuation of these studies, the structure of the title compound (I) is described (Fig. 1). Bond lengths and angles, can be regarded as typical for *N*,*N'*-disubstituted thiourea compounds as found in the Cambridge Structural Database v5.28 (Allen, 2002; Khawar Rauf *et al.*, 2006*b*; Arslan *et al.*, 2004; Yamin & Yusof, 2003).The molecule exists in the thione form with typical thiourea C—S and C—O bonds, as well as shortened C—N bond lengths. The thiocarbonyl and carbonyl groups are almost coplanar. The molecule features an intramolecular N—H···O hydrogen bond and in the crystal structure, molecules associate *via* N—H···S intermolecular hydrogen bonds to form centrosymmetric dimers (Table 1; Fig 2).In addition to the intramolecular hydrogen bond, O1 is involved in a short O···Cl contact [O1···Cl2^i^: 3.0936 (14) Å, symmetry operator i: 1 - *x*, 1 - *y*, 1 - *z*].

### Experimental

Freshly prepared 2,6-dichlorobenzoyl isothiocyanate (2.32 g, 10 mmol) was stirred in acetone (40 ml) for 20 minutes. Neat 3,5-dichloroaniline (1.62 g, 10 mmol) was then added and the resulting mixture was stirred for 1 h. The reaction mixture was then poured into acidified (pH 4) water and stirred well. The solid product was separated and washed with deionized water and purified by recrystallization from methanol/ 1,1-dichloromethane (1:10 *v/v*) to give fine crystals of (I), with an overall yield of 80%.

### Refinement

Hydrogen atoms bonded to C were included in calculated positions and refined as riding on their parent C atom with C—H = 0.95 Å *U*_iso_(H) = 1.2*U*_eq_(C). The H atoms bonded to N were freely refined.

### Crystal data

**Table:**

C_14_H_8_Cl_4_N_2_OS	*F*(000) = 792
*M**_r_* = 394.08	*D*_x_ = 1.610 Mg m^−^^3^
Monoclinic, *P*2_1_/*c*	Mo *K*α radiation, λ = 0.71073 Å
Hall symbol: -P 2ybc	Cell parameters from 13230 reflections
*a* = 14.7737 (13) Å	θ = 3.8–27.8°
*b* = 10.3744 (6) Å	µ = 0.86 mm^−^^1^
*c* = 10.6935 (11) Å	*T* = 173 K
β = 97.250 (7)°	Plate, colourless
*V* = 1625.9 (2) Å^3^	0.42 × 0.38 × 0.21 mm
*Z* = 4	

### Data collection

**Table:**

Stoe IPDSII two-circle diffractometer	3724 independent reflections
Radiation source: fine-focus sealed tube	3306 reflections with *I* > 2σ(*I*)
graphite	*R*_int_ = 0.046
ω scans	θ_max_ = 27.5°, θ_min_ = 3.7°
Absorption correction: multi-scan (*MULABS*; Spek, 2003; Blessing, 1995)	*h* = −19→19
*T*_min_ = 0.715, *T*_max_ = 0.841	*k* = −13→13
12823 measured reflections	*l* = −10→13

### Refinement

**Table:**

Refinement on *F*^2^	Secondary atom site location: difference Fourier map
Least-squares matrix: full	Hydrogen site location: inferred from neighbouring sites
*R*[*F*^2^ > 2σ(*F*^2^)] = 0.032	H atoms treated by a mixture of independent and constrained refinement
*wR*(*F*^2^) = 0.087	*w* = 1/[σ^2^(*F*_o_^2^) + (0.0525*P*)^2^ + 0.486*P*] where *P* = (*F*_o_^2^ + 2*F*_c_^2^)/3
*S* = 1.05	(Δ/σ)_max_ = 0.001
3724 reflections	Δρ_max_ = 0.44 e Å^−^^3^
208 parameters	Δρ_min_ = −0.36 e Å^−^^3^
0 restraints	Extinction correction: *SHELXL97* (Sheldrick, 2008), Fc^*^=kFc[1+0.001xFc^2^λ^3^/sin(2θ)]^-1/4^
Primary atom site location: structure-invariant direct methods	Extinction coefficient: 0.0246 (15)

### Special details

**Table:**

Geometry. All e.s.d.'s (except the e.s.d. in the dihedral angle between two l.s. planes) are estimated using the full covariance matrix. The cell e.s.d.'s are taken into account individually in the estimation of e.s.d.'s in distances, angles and torsion angles; correlations between e.s.d.'s in cell parameters are only used when they are defined by crystal symmetry. An approximate (isotropic) treatment of cell e.s.d.'s is used for estimating e.s.d.'s involving l.s. planes.
Refinement. Refinement of *F*^2^ against ALL reflections. The weighted *R*-factor *wR* and goodness of fit *S* are based on *F*^2^, conventional *R*-factors *R* are based on *F*, with *F* set to zero for negative *F*^2^. The threshold expression of *F*^2^ > σ(*F*^2^) is used only for calculating *R*-factors(gt) *etc*. and is not relevant to the choice of reflections for refinement. *R*-factors based on *F*^2^ are statistically about twice as large as those based on *F*, and *R*- factors based on ALL data will be even larger.

### Fractional atomic coordinates and isotropic or equivalent isotropic displacement parameters (Å^2^)

**Table:**

	*x*	*y*	*z*	*U*_iso_*/*U*_eq_	
Cl1	0.35875 (3)	0.16745 (4)	0.09234 (4)	0.02939 (13)	
Cl2	0.39163 (3)	0.58457 (4)	0.39696 (4)	0.02796 (12)	
Cl3	0.93189 (3)	0.14605 (4)	0.52460 (4)	0.03065 (13)	
Cl4	0.97033 (3)	0.59117 (4)	0.27286 (4)	0.02639 (12)	
C1	0.46669 (10)	0.35599 (15)	0.26849 (14)	0.0171 (3)	
O1	0.50255 (7)	0.29825 (13)	0.36158 (11)	0.0257 (3)	
N1	0.51344 (8)	0.40910 (14)	0.17722 (13)	0.0185 (3)	
H1	0.4844 (15)	0.443 (2)	0.115 (2)	0.028 (5)*	
S1	0.64669 (2)	0.48065 (5)	0.04928 (4)	0.02559 (13)	
C2	0.60770 (10)	0.41647 (15)	0.17586 (15)	0.0168 (3)	
N2	0.65773 (8)	0.36848 (14)	0.27949 (13)	0.0178 (3)	
H2	0.6248 (15)	0.327 (2)	0.333 (2)	0.033 (6)*	
C11	0.36443 (10)	0.37608 (15)	0.24447 (14)	0.0170 (3)	
C12	0.30851 (10)	0.29239 (16)	0.16745 (15)	0.0196 (3)	
C13	0.21408 (11)	0.30756 (19)	0.14814 (18)	0.0265 (4)	
H13	0.1771	0.2506	0.0941	0.032*	
C14	0.17491 (11)	0.40754 (19)	0.2093 (2)	0.0300 (4)	
H14	0.1105	0.4177	0.1978	0.036*	
C15	0.22860 (12)	0.49305 (18)	0.28705 (18)	0.0270 (4)	
H15	0.2014	0.5610	0.3288	0.032*	
C16	0.32308 (10)	0.47701 (16)	0.30247 (15)	0.0195 (3)	
C21	0.75373 (10)	0.37152 (16)	0.31296 (14)	0.0170 (3)	
C22	0.79148 (10)	0.27196 (16)	0.39134 (15)	0.0196 (3)	
H22	0.7539	0.2054	0.4175	0.024*	
C23	0.88510 (10)	0.27226 (16)	0.43034 (15)	0.0208 (3)	
C24	0.94227 (10)	0.36829 (17)	0.39447 (15)	0.0217 (3)	
H24	1.0062	0.3666	0.4205	0.026*	
C25	0.90178 (10)	0.46720 (16)	0.31857 (15)	0.0189 (3)	
C26	0.80870 (10)	0.47105 (16)	0.27637 (15)	0.0184 (3)	
H26	0.7832	0.5394	0.2242	0.022*	

### Atomic displacement parameters (Å^2^)

**Table:**

	*U*^11^	*U*^22^	*U*^33^	*U*^12^	*U*^13^	*U*^23^
Cl1	0.0285 (2)	0.0291 (2)	0.0314 (2)	−0.00172 (16)	0.00678 (17)	−0.00990 (17)
Cl2	0.0319 (2)	0.0247 (2)	0.0269 (2)	−0.00439 (15)	0.00228 (17)	−0.00444 (16)
Cl3	0.0275 (2)	0.0330 (2)	0.0291 (2)	0.00629 (16)	−0.00562 (16)	0.01025 (18)
Cl4	0.01753 (19)	0.0297 (2)	0.0324 (2)	−0.00597 (14)	0.00488 (15)	0.00171 (16)
C1	0.0145 (6)	0.0204 (8)	0.0165 (7)	−0.0021 (5)	0.0017 (5)	0.0004 (6)
O1	0.0169 (5)	0.0394 (7)	0.0207 (6)	−0.0008 (5)	0.0015 (4)	0.0116 (5)
N1	0.0121 (6)	0.0269 (7)	0.0160 (6)	0.0004 (5)	0.0005 (5)	0.0066 (5)
S1	0.01402 (18)	0.0436 (3)	0.0194 (2)	0.00106 (16)	0.00325 (14)	0.01158 (17)
C2	0.0135 (6)	0.0201 (8)	0.0168 (7)	0.0008 (5)	0.0018 (5)	0.0010 (6)
N2	0.0120 (6)	0.0237 (7)	0.0177 (6)	−0.0012 (5)	0.0014 (5)	0.0037 (5)
C11	0.0138 (6)	0.0216 (8)	0.0158 (7)	−0.0009 (5)	0.0032 (5)	0.0048 (6)
C12	0.0172 (7)	0.0226 (8)	0.0192 (8)	−0.0013 (6)	0.0035 (5)	0.0014 (6)
C13	0.0169 (7)	0.0316 (9)	0.0298 (9)	−0.0065 (6)	−0.0021 (6)	0.0016 (7)
C14	0.0135 (7)	0.0371 (10)	0.0391 (10)	0.0025 (7)	0.0016 (7)	0.0051 (8)
C15	0.0222 (8)	0.0267 (9)	0.0330 (10)	0.0065 (6)	0.0065 (7)	0.0029 (7)
C16	0.0191 (7)	0.0205 (8)	0.0186 (7)	−0.0009 (6)	0.0015 (6)	0.0028 (6)
C21	0.0126 (6)	0.0226 (8)	0.0157 (7)	0.0014 (5)	0.0009 (5)	−0.0016 (6)
C22	0.0178 (7)	0.0232 (8)	0.0178 (7)	−0.0002 (6)	0.0016 (5)	0.0013 (6)
C23	0.0213 (7)	0.0244 (8)	0.0157 (7)	0.0050 (6)	−0.0010 (6)	0.0017 (6)
C24	0.0142 (6)	0.0298 (9)	0.0204 (8)	0.0020 (6)	−0.0009 (5)	−0.0026 (7)
C25	0.0150 (7)	0.0242 (8)	0.0178 (7)	−0.0024 (6)	0.0027 (5)	−0.0019 (6)
C26	0.0147 (6)	0.0213 (8)	0.0188 (7)	0.0007 (5)	0.0011 (5)	0.0005 (6)

### Geometric parameters (Å, °)

**Table:**

Cl1—C12	1.7397 (17)	C13—C14	1.390 (3)
Cl2—C16	1.7417 (17)	C13—H13	0.9500
Cl3—C23	1.7414 (17)	C14—C15	1.393 (3)
Cl4—C25	1.7445 (16)	C14—H14	0.9500
C1—O1	1.224 (2)	C15—C16	1.395 (2)
C1—N1	1.3796 (19)	C15—H15	0.9500
C1—C11	1.5146 (19)	C21—C26	1.400 (2)
N1—C2	1.3967 (18)	C21—C22	1.401 (2)
N1—H1	0.82 (2)	C22—C23	1.393 (2)
S1—C2	1.6745 (16)	C22—H22	0.9500
C2—N2	1.347 (2)	C23—C24	1.391 (2)
N2—C21	1.4186 (18)	C24—C25	1.395 (2)
N2—H2	0.91 (2)	C24—H24	0.9500
C11—C12	1.394 (2)	C25—C26	1.393 (2)
C11—C16	1.396 (2)	C26—H26	0.9500
C12—C13	1.393 (2)		
			
O1—C1—N1	124.54 (14)	C14—C15—C16	118.71 (16)
O1—C1—C11	121.70 (13)	C14—C15—H15	120.6
N1—C1—C11	113.75 (13)	C16—C15—H15	120.6
C1—N1—C2	128.18 (14)	C15—C16—C11	121.63 (15)
C1—N1—H1	118.9 (15)	C15—C16—Cl2	119.48 (13)
C2—N1—H1	112.9 (15)	C11—C16—Cl2	118.89 (12)
N2—C2—N1	114.54 (13)	C26—C21—C22	120.66 (14)
N2—C2—S1	127.07 (11)	C26—C21—N2	122.87 (14)
N1—C2—S1	118.38 (11)	C22—C21—N2	116.38 (14)
C2—N2—C21	128.79 (13)	C23—C22—C21	118.86 (15)
C2—N2—H2	114.5 (15)	C23—C22—H22	120.6
C21—N2—H2	116.7 (15)	C21—C22—H22	120.6
C12—C11—C16	118.01 (13)	C24—C23—C22	122.24 (15)
C12—C11—C1	121.24 (14)	C24—C23—Cl3	119.19 (12)
C16—C11—C1	120.72 (14)	C22—C23—Cl3	118.57 (13)
C13—C12—C11	121.66 (15)	C23—C24—C25	117.16 (14)
C13—C12—Cl1	119.56 (13)	C23—C24—H24	121.4
C11—C12—Cl1	118.78 (11)	C25—C24—H24	121.4
C14—C13—C12	118.88 (16)	C26—C25—C24	122.87 (15)
C14—C13—H13	120.6	C26—C25—Cl4	118.19 (13)
C12—C13—H13	120.6	C24—C25—Cl4	118.93 (12)
C13—C14—C15	121.08 (15)	C25—C26—C21	118.19 (14)
C13—C14—H14	119.5	C25—C26—H26	120.9
C15—C14—H14	119.5	C21—C26—H26	120.9
			
O1—C1—N1—C2	−4.9 (3)	C14—C15—C16—Cl2	178.83 (14)
C11—C1—N1—C2	174.63 (15)	C12—C11—C16—C15	1.5 (2)
C1—N1—C2—N2	−2.2 (2)	C1—C11—C16—C15	−176.47 (15)
C1—N1—C2—S1	177.27 (14)	C12—C11—C16—Cl2	−178.89 (12)
N1—C2—N2—C21	−173.75 (15)	C1—C11—C16—Cl2	3.2 (2)
S1—C2—N2—C21	6.9 (3)	C2—N2—C21—C26	30.3 (3)
O1—C1—C11—C12	−95.3 (2)	C2—N2—C21—C22	−152.93 (16)
N1—C1—C11—C12	85.17 (19)	C26—C21—C22—C23	−1.2 (2)
O1—C1—C11—C16	82.6 (2)	N2—C21—C22—C23	−178.08 (14)
N1—C1—C11—C16	−96.94 (18)	C21—C22—C23—C24	0.2 (2)
C16—C11—C12—C13	−0.1 (2)	C21—C22—C23—Cl3	−178.45 (12)
C1—C11—C12—C13	177.83 (15)	C22—C23—C24—C25	1.1 (2)
C16—C11—C12—Cl1	179.03 (12)	Cl3—C23—C24—C25	179.77 (12)
C1—C11—C12—Cl1	−3.0 (2)	C23—C24—C25—C26	−1.5 (2)
C11—C12—C13—C14	−1.1 (3)	C23—C24—C25—Cl4	179.11 (12)
Cl1—C12—C13—C14	179.72 (14)	C24—C25—C26—C21	0.5 (2)
C12—C13—C14—C15	1.1 (3)	Cl4—C25—C26—C21	179.94 (12)
C13—C14—C15—C16	0.2 (3)	C22—C21—C26—C25	0.9 (2)
C14—C15—C16—C11	−1.5 (3)	N2—C21—C26—C25	177.49 (14)

### Hydrogen-bond geometry (Å, °)

**Table:**

*D*—H···*A*	*D*—H	H···*A*	*D*···*A*	*D*—H···*A*
N2—H2···O1	0.91 (2)	1.89 (2)	2.6581 (17)	141 (2)
N1—H1···S1^i^	0.82 (2)	2.57 (2)	3.3653 (14)	163 (2)

Symmetry codes: (i) −*x*+1, −*y*+1, −*z*.
